# Single-cell transcriptomics and functional validation revealed PLEKHA5-L as a promoter of growth and migration in brain metastatic melanoma cells

**DOI:** 10.3389/fonc.2025.1560954

**Published:** 2025-04-28

**Authors:** Xiaogen Tang, Tingting Lei, Boya Huang, Guangjie Wu, Yizhen Tian, Jian Xiang, Dongwei Fu, Hongyi Zhang

**Affiliations:** ^1^ Department of Microbiology and Immunology, School of Medicine, Jinan University, Guangzhou, Guangdong, China; ^2^ Department of Systems Biomedical Sciences, School of Medicine, Jinan University, Guangzhou, Guangdong, China; ^3^ Department of Oncology, The Affiliated Shunde Hospital of Jinan University, Jinan University, Foshan, Guangdong, China

**Keywords:** melanoma, brain metastasis, PLEKHA5, single-cell RNA sequencing, PLEKHA-L

## Abstract

**Background:**

Melanoma brain metastasis is an lethal event. Investigating the molecules that potentially promoted melanoma metastasis is important for targeted therapy.

**Methods:**

The transcriptional profiles of totaling 7 melanoma samples, including 4 primary and 3 brain metastatic tissues were studied on the single-cell RNA sequencing level, and the expression of PLEKHA5 was examined in tumor clusters. Then PLEKHA5 expression was validated in brain Metastatic model by left ventricular injections in nude mice. The functional effect of PLEKHA5 isoforms (Long or Short) on proliferation and migration of melanoma was studied by RNA interference, overexpression by lentivirus vector, CCK8 test, colony formation test, transwell chamber assay. The targets and signal pathways that was potentially regulated by PLEKHA5 was studied by RNA-sequencing.

**Result:**

PLEKHA5 expression increased in brain metastatic melanoma at single cell level. PLEKH5 was constantly upregulated in brain metastatic tissue of melanoma in animal model. PLEKHA5-L had the potential for melanoma migration and proliferation by upregulating oncogenes such as HRAS, AKT3 etc. PLEKHA5-L also upregulated expression of PD-L1 and ABC transporters that were associated with therapy resistant.

**Conclusion:**

PLEKHA5-L was potential therapeutic target for metastatic melanoma.

## Introduction

The brain is a common site for melanoma metastasis, ranking as the third most frequent cancer to affect the central nervous system, following lung and breast cancers. It is estimated that 40-50% of stage IV melanoma patients will develop clinically detectable brain metastases. Autopsies reveal that over 70% of patients have brain metastases, indicating a high rate of subclinical occurrences (1–3). Untreated melanoma brain metastasis (MBM) progresses rapidly, typically reducing survival time to about 3 months; brain metastases are responsible for roughly 50% of melanoma-related deaths (3).

The process by which melanoma cells metastasized to the brain involved hematogenous spread, favoring areas with the highest blood flow, such as the cerebral hemispheres (4). The molecular mechanisms behind this metastasis were not fully understood and were thought to involve a complex, multi-step process. Initially, primary tumor cells invaded local tissues and entered the bloodstream, disseminating until they were trapped in the capillary beds of organs, resulting in metastases (5, 6). Adhesion of tumor cells to the brain endothelium was a crucial early step, potentially mediated by molecules like the chemokine receptor 4 (CCR4), which triggered the PI3K/AKT pathway upon binding (7). As metastases grew, melanoma cells promoted angiogenesis to obtain nutrients from adjacent healthy tissue. The JAK/STAT pathway, especially the continuous activation of STAT3, was involved, enhancing the expression of angiogenic factors such as VEGF, MMP2, and FGF2. These factors facilitated invasion and neovascularization, thereby fueling the growth of metastatic tumors (8).

Despite extensive research on melanoma brain metastases, the underlying mechanisms remained unclear. Identifying novel biomarkers and gaining a deeper understanding of MBM’s molecular biology were crucial for developing targeted therapies. The existing gap in knowledge posed a considerable challenge to advancing treatments for this aggressive condition.

PLEKHA5, a member of the PLEKHA family characterized by its pleckstrin homology (PH) domains, additionally featured Trp-Trp (WW) motifs. These PH domains interacted with phosphoinositides, participating in cellular processes such as signal transduction and vesicular transport. The PLEKHA family, devoid of catalytic activity, served as adaptor proteins (9). Multiple PLEKHA5 splice variants existed in humans and mice, their functions being under investigation at the time (10).

The long isoform of PLEKHA5 (PLEKHA5-L), representing the predominant variant in adulthood, comprises 32 exons encoding a 1282-amino acid protein that exhibits brain-specific expression patterns. In contrast, the short isoform PLEKHA5-S arises through alternative splicing of PLEKHA5-L, containing 26 exons that encode a truncated 1116-amino acid polypeptide with ubiquitous tissue distribution. Both isoforms demonstrate functional importance in normative cerebral development. Biochemical characterization reveals that the full-length PLEKHA5 localizes to the plasma membrane and maintains microtubule association through its C-terminal PDZ-binding motif (10).

PLEKHA5 has been extensively implicated in tumorigenesis and cancer progression through context-dependent mechanisms. In melanoma, PLEKHA5 was initially reported to be highly overexpressed compared to normal tissues (9). Subsequent investigations linked its expression to early-stage brain metastasis development, demonstrating that PLEKHA5 inhibition suppresses melanoma cell proliferation and invasion in both intracranial and extracranial microenvironments (11). Recent mechanistic studies reveal that PLEKHA5 accelerates brain metastatic growth by activating the AKT pathway to promote PDCD4 degradation. This process counteracts PDCD4-mediated cell cycle suppression via upregulated p27 and p21 expression, ultimately driving tumor progression (12). Similar oncogenic roles are observed in MET-amplified diffuse gastric cancer, where PLEKHA5 silencing inhibits the growth of MET inhibitor-resistant tumor cells (13). Paradoxically, PLEKHA5 exhibits tumor-suppressive effects in breast cancer metastasis, with knockout models displaying enhanced migratory and invasive capacities (14). The molecular basis for these cancer-type-specific discrepancies remains elusive. Current hypotheses suggest either tissue-dependent functional plasticity of PLEKHA5 or divergent roles of its splice isoforms in tumorigenic processes (13).

Although existing studies have established the pro-metastatic role of PLEKHA5 in melanoma brain metastasis, its precise biological functions and molecular mechanisms during this process remain unclear. Importantly, whether distinct splicing isoforms of PLEKHA5 (primarily PLEKHA5-L and PLEKHA5-S) exert differential effects on brain metastatic niche formation requires further investigation. To comprehensively characterize the roles of these two major isoforms in melanoma brain metastasis, we will combine molecular, cellular, and orthotopic animal models to systematically evaluate their functions through isoform-specific knockdown and overexpression approaches. This multi-level strategy will dissect PLEKHA5’s contributions to metastasis initiation, circulatory spread, and brain parenchyma colonization. Furthermore, our exploration of the underlying mechanisms aims to provide a theoretical foundation for developing clinical therapies targeting melanoma brain metastasis.

Our findings demonstrated that the PLEKHA5-L isoform plays a more profound role in melanoma brain metastasis initiation and progression. PLEKHA5-L upregulates oncogenes (e.g., HRAS, AKT3) to enhance melanoma migration and proliferation, while concomitantly elevating the expression of therapy-resistance mediators PD-L1 and ABC transporters. These multi-dimensional effects establish PLEKHA5-L as a potential therapeutic target in metastatic melanoma.

## Methods

### Human specimen data sets

This study data consists of 2 data sets. The data discussed in this publication have been deposited in NCBI's Gene Expression Omnibus and are accessible through GEO Series accession number GSE139829, GSE186344. Ultimately, we obtained 7 scRNA-seq samples. including 4 primary (P) and 3 brain metastatic (BM) tissues (**Supplementary Table 1**).

### Single-cell sequencing data processing

scRNA-seq FASTQ files were processed with Human reference genome GRCh38-3.0.0 by the 10X Genomics Cell Ranger software (v6.0.0) with default parameter settings. R package Seurat (v4.3.0) was used for further analysis. Cells from each sample with nFeature_RNA > 200, nFeature_RNA < 7500 and mitochondria gene percentage < 10 were kept for downstream analysis. Expression matrices of the 7 samples were merged into a data object with FindIntegrationAnchors and IntegrateData. The initial dataset contained 33,133 cells. For the combined dataset, the clustering resolution was set to 0.2. Marker genes for each significant cluster were found using the FindAllMarkers function. Differentially expressed genes in clusters and cell types are summarized in **Supplementary Table 1**.

### Pan-cancer and survival analysis

The TCGA pan-cancer data was used to evaluate the prognostic effects of PLEKHA5 expression. The gene expression and clinical data were downloaded from UCSC Xena (https://xena.ucsc.edu/). We investigated the expression levels of PLEKHA5 in 33 normal tissues and corresponding tumors. According to the median expression of PLEKHA5, the patients were divided into high and low expression groups for survival analysis. Survminer (v0.4.9) was used to plot the OS curves. Paired tumor and normal data from the GTEx database were also included in the analysis.

#### Quantization and data analysis

Statistics are calculated using Wilcoxon’s rank sum test. P-values less than 0.05 were considered significant and were marked as follows: P < 0.05 (*), P < 0.01 (**) and P < 0.001 (***).

### Data availability

The data discussed in this publication have been deposited in NCBI's Gene Expression Omnibus and are accessible through GEO Series accession number GSE139829, GSE186344.

### Cell lines

JUN-M1(M1) cells, A2058 cells and HEK293T cells were cultured in DMEM medium (KeyGEN, KG1206), supplemented with 10% fetal bovine serum (FBS) (ExCell, FSD500) and 1% penicillin/streptomycin. The JUN-M1 cells with/out PLEKHA5-L, PLEKHA5-S overexpression and JUN-M1-Brm cells with/out PLKHA5 knockdown were cultured in DMEM medium supplemented with 10% fetal bovine serum. All cell culture experiments were conducted at 37°C in an atmosphere of 5% CO2. HEK293T cells were utilized to produce lentivirus. Upon reaching near confluence, the cells were detached using 0.25% (w/v) Trypsin-EDTA and then either sub-cultured or used for experimentation.

### Transwell migration assays

The cell pellet was resuspended in serum-free medium and adjusted to optimal density for counting.Transwell chambers (Corning,#3422) were placed in 24-well plates. Cells (4×10⁴/200 μL serum-free medium) were seeded into the upper chambers, while the lower chambers were filled with 500 μL complete DMEM medium. After 24 h, chambers were rinsed with PBS. Migrated cells on the lower membrane were fixed with methanol (15 min) and stained with 0.5% crystal violet (15 min). Non-migrated cells were removed via cotton swab.

Cell counts were performed under an inverted microscope (five random fields/chamber, 100×).

### Animal experiment

All experimental procedures were approved by the Jinan University Animal Ethics Committee. Primary JUN-M1 cells were transfected with luciferase-expressing plasmid pLenti CMV V5-LUC Blast. For intracardiac Injection, anesthetized 6 weeks old nude mice received intracardiac injections of 2×10^⁵^ cells per 200 μL ice-cold PBS using a 27-28G needle. Successful left ventricular entry was confirmed by bright blood reflux. Metastasis was monitored using IVIS 200 after D-Luciferin(PerkinElmer, #122799) injection(20 μL). Metastatic lesions were analyzed at 3 weeks post-injection.

### Plasmid construction

We constructed the human PLEKHA5-L/S gene onto the PLVX-Neo vector. The CDS region of PLEKHA5-L/S was amplified from pCDH-CMV-PLEKHA5-EF1-CopGFP-T2A-Puro and pLV3-CMV-PLEKHA5 (human)-CopGFP-Puro vector and then inserted into the vector using the ClonExpress MultiS One Step Cloning Kit (Vazyme Biotech, #C113-02), which is based on in vitro multi-fragment recombination. The oligo primer sequences for PLEKHA5-L/S DNA amplification are as follows (5’-3’):

Forward Primer: CGAGCTCAAGCTTCGATGGCGGCGGATCTGAACCT. Reverse Primer: TAGAGTCGCGGGATCCTACACACACATGAAATGTG. To construct the PLEKHA5 knock-down recombinant plasmid The shPLEKHA5 with the target sequence 5'-CCCGCTACCCTGAAGGTTATA-3' and MISSION pLKO.1-puro control were purchased form (Miaoling Biology,# P56553). T4 DNA Ligase (NEB, #M0202S) linked the annealed shRNA duplex to it.

### Lentiviral transduction and the generation of stable cell lines

Lentiviruses were produced by transfecting HEK293T cells with the shRNA-targeting plasmids and the helper plasmids psPAX2 and pMD2.G. The cell supernatants were collected 72 hours after transfection and were either used immediately to infect cells or stored at -80°C. To obtain stable cell lines, cells were infected at 20% confluence for 24 hours with lentiviral supernatants diluted 2:1 with normal culture medium in the presence of 8 ug/mL of polybrene (Beyotime, #C0351). At 48 hours after infection, the cells were subjected to puromycin or G418 selection at a concentration of 2 μg/mL for 1-2 week before being passaged for use.

### Cell proliferation and colony formation assays

To assess cell proliferation in vitro, the Cell Counting Kit-8 (CCK8, Beyotime, #C0039) assay was performed according to the manufacturer's instructions. Flat-bottom 96-well plates were seeded with 400 cells per well and cultured for 7 days. For the CCK8 assay, 10 µL of CCK8 solution was added to the cells in the 96-well plates, followed by a 2-hour incubation at 37°C. Absorbance was measured at 450 nm.

For colony formation assays, 200 cells per well were plated in 6 cm dishes and cultured for 14 days. The dishes were washed with PBS twice and then fixed with methanol for 10 minutes. Subsequently, the cell colonies were stained with 0.5% crystal violet (in ethanol) for 15 minutes.

### Total RNA extraction and NGS RNA-sequence

Total RNA was isolated from samples using TRIzol Reagent (Invitrogen, #15596-026) according to the manufacturer's instructions. The quality was assessed with a NanoDrop 2000 spectrophotometer (Thermo Scientific, USA), ensuring the 260/280 wavelength ratio was within the range of 1.9 to 2.0. The extracted RNA was shipped on dry ice to Novogene for sequencing. The sequencing data in FASTQ format were aligned to the human reference genome Homo_sapiens_hg38 using TopHat (v2.1.1). Differential expression analysis of genes was performed using DESeq2 (v1.32.0), with differentially expressed genes (DEGs) selected based on an adjusted p-value (padj) < 0.05 and a fold change (FC) < 0.5 or > 2. To assess the quality of the sequencing data, principal component analysis (PCA) was conducted to evaluate the distribution of samples. Gene Ontology (GO) analysis and Kyoto Encyclopedia of Genes and Genomes (KEGG) pathway enrichment were performed on the gene list using the ClusterProfiler R package (v4.0.5), with a padj value < 0.05 considered to indicate significant enrichment.

### Western blot

Cells were washed twice with PBS. Ice-cold RIPA lysis buffer (Beyotime, #P0013B), containing fresh PMSF (100 mM) (Beyotime, #ST506), was added to the cells and incubated for 20 minutes on ice. Cells were harvested into centrifuge tubes and vortexed vigorously to ensure complete lysis. Following this, centrifugation was performed at 14,000 × g for 10 minutes at 4°C. Cell lysate protein concentrations were determined using the BCA Protein Assay Kit (Thermo Fisher Scientific, #23225). Thirty micrograms of total protein samples were separated by electrophoresis on a 10% SDS-PAGE gel and electro-transferred for 120 minutes onto PVDF membranes. After sealing with 5% skimmed milk for 60 minutes, the appropriate primary antibodies (β-Actin: Bimake, # A5108 , # PLEKHA5:, #) were added and incubated overnight at 4°C. Membranes were washed five times with Tris-buffered saline containing 0.1% Tween 20 (TBST) and then incubated with an HRP-conjugated secondary antibody (1:5000) at room temperature for 60 minutes. After washing the membranes again, protein levels were detected using an enhanced chemiluminescence (ECL) kit (Fude, #FD8020). Protein expression levels were normalized to β-actin. The ImageJ software was used to quantify the band density.

### Quantization and data analysis

Statistics are calculated using t test, one-way ANOVA and Wilcoxon’s rank sum test. GraphPad Prism 8 was used for data processing and plotting. P-values less than 0.05 were considered significant and were marked as follows: P < 0.05 (*), P < 0.01 (**) and P < 0.001 (***).

## Results

### Single cell atlas of melanoma samples

**Figure 1:**
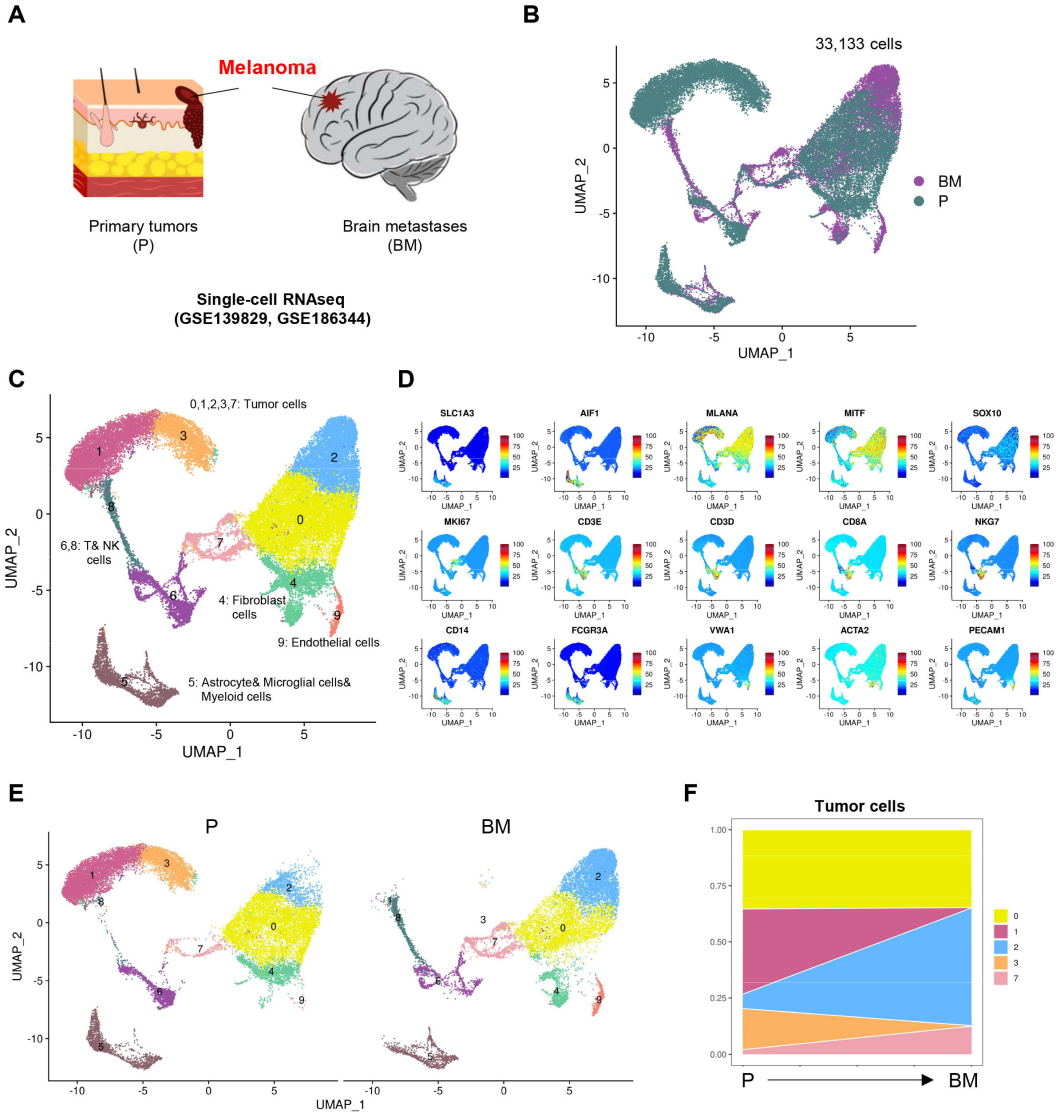
Single cell atlas of melanoma samples. **(A)** Schematic diagram of melanoma sample collection for scRNA-seq. **(B)** UMAP (Uniform manifold approximation and projection) visualization of single cell data from all samples colored by group (Primary tumors (P), Brain metastases (BM)). **(C)** UMAP visualization of single cell data from all samples colored by clusters. **(D)** Projection of expression levels of selected marker genes on UMAP visualization. **(E)** UMAP visualization of single cell data clusters split by P and BM. **(F)** Variation of proportion of each tumor cell clusters through melanoma from P to BM.

Melanoma is one of the most likely malignant tumors to metastasize to the brain, with up to 60% of melanoma patients experiencing melanoma brain metastasis (MBM). We collected single-cell transcriptomic data from two public datasets, totaling 7 melanoma samples, including 4 primary (P) and 3 brain metastatic (BM) tissues (**Figure 1A**). After quality control and integration of these single-cell transcriptomic samples, we obtained a total of 33,133 high-quality cells, comprising 20,111 cells from primary and 13,022 cells from brain metastatic, with no obvious batch effects observed between the samples (**Figure 1B**). Upon clustering the integrated data, we identified a total of 10 cell clusters, defined by marker genes characteristic of different cell populations (**Figures 1C, D**). Among these, clusters 0, 1, 2, 3, and 7 are tumor cells that highly express MLANA, MITF, SOX10, and MKI67. Cluster 4 consisted of fibroblasts that highly express ACTA2, while cluster 5 was a large group of astrocytes, microglial cells, and myeloid cells, expressing both the astrocyte marker SLC1A3 and the microglial marker AIF1, as well as the myeloid cell markers CD14 and FCGR3A. Clusters 6 and 8 are T and NK cells, while cluster 9 consists of endothelial cells that highly express VWA1 and PECAM1.

Next, we examined the differences in these cell clusters between primary and brain metastatic groups. We found that for tumor cells, the proportion of cluster 0 did not differ significantly between the primary and brain metastatic samples; however, cluster 1 and 3 were predominantly present in the primary samples, while cluster 2 and 7 were more prevalent in the brain metastatic samples (**Figures 1E, F**). We hold opinion that cluster 2 and 7 represent unique tumor cell populations that specifically arise in brain metastasis.

### The expression of PLEKHA5 in tumor cells and pan-cancer

**Figure 2:**
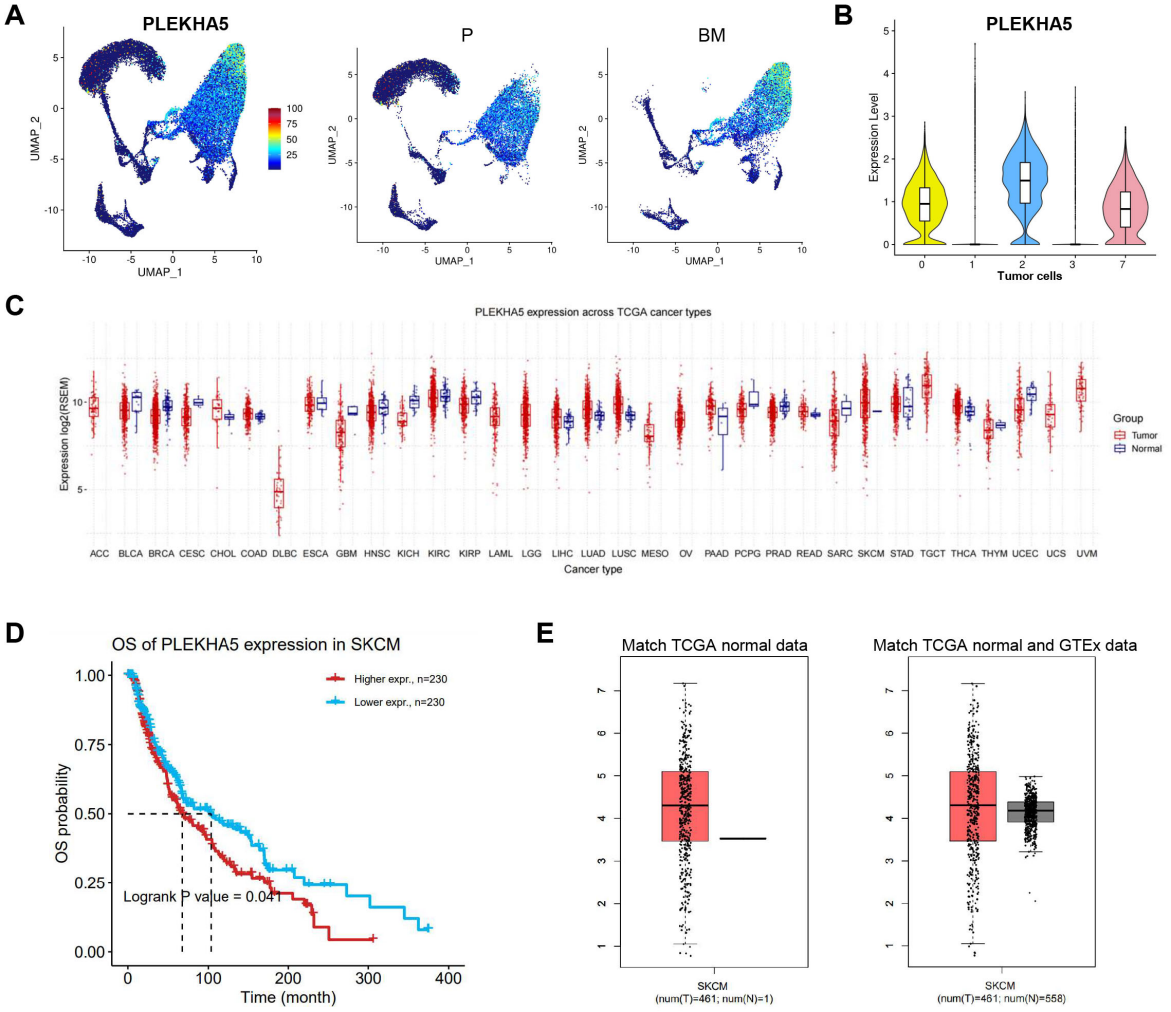
The expression of PLEKHA5 in tumor cells and pan-cancer. **(A)** Left: The expression levels of PLEKHA5 on UMAP visualization. Right: The expression levels of PLEKHA5 on UMAP visualization split by P and BM. **(B)** VlnPlot of the expression levels of PLEKHA5 in all tumor cells. **(C)** Boxplots for pan-cancer PLEKHA5 expression comparison. **(D)** The OS of PLEKHA5 expression in SKCM. **(E)** Boxplots for PLEKHA5 expression comparison in SKCM, both match TCGA normal data and match TCGA normal and GTEx data.

Next, we examined the expression of PLEKHA5 in melanoma and compared the differences in PLEKHA5 expression between primary and brain metastatic groups (**Figure 2A**). We found that PLEKHA5 was highly expressed in tumor cell cluster 0, 2, and 7, indicating that PLEKHA5 is upregulated in melanoma brain metastasis samples (**Figure 2B**).

Subsequently, we analyzed the expression of PLEKHA5 in normal versus tumor samples across different cancer types in the TCGA database. The expression levels varied among different cancer types; in most cases, tumor samples exhibited higher expression than normal samples, particularly in SKCM (**Figure 2C**). Additionally, the high expression of PLEKHA5 in melanoma was associated with poor prognosis (**Figure 2D**). We found that PLEKHA5 expression in tumor samples was higher than in normal samples in both matched TCGA normal data and matched TCGA normal and GTEx data (**Figure 2E**).

### PLEKHA5 expression increased along with brain metastatic melanoma cell

**Figure 3:**
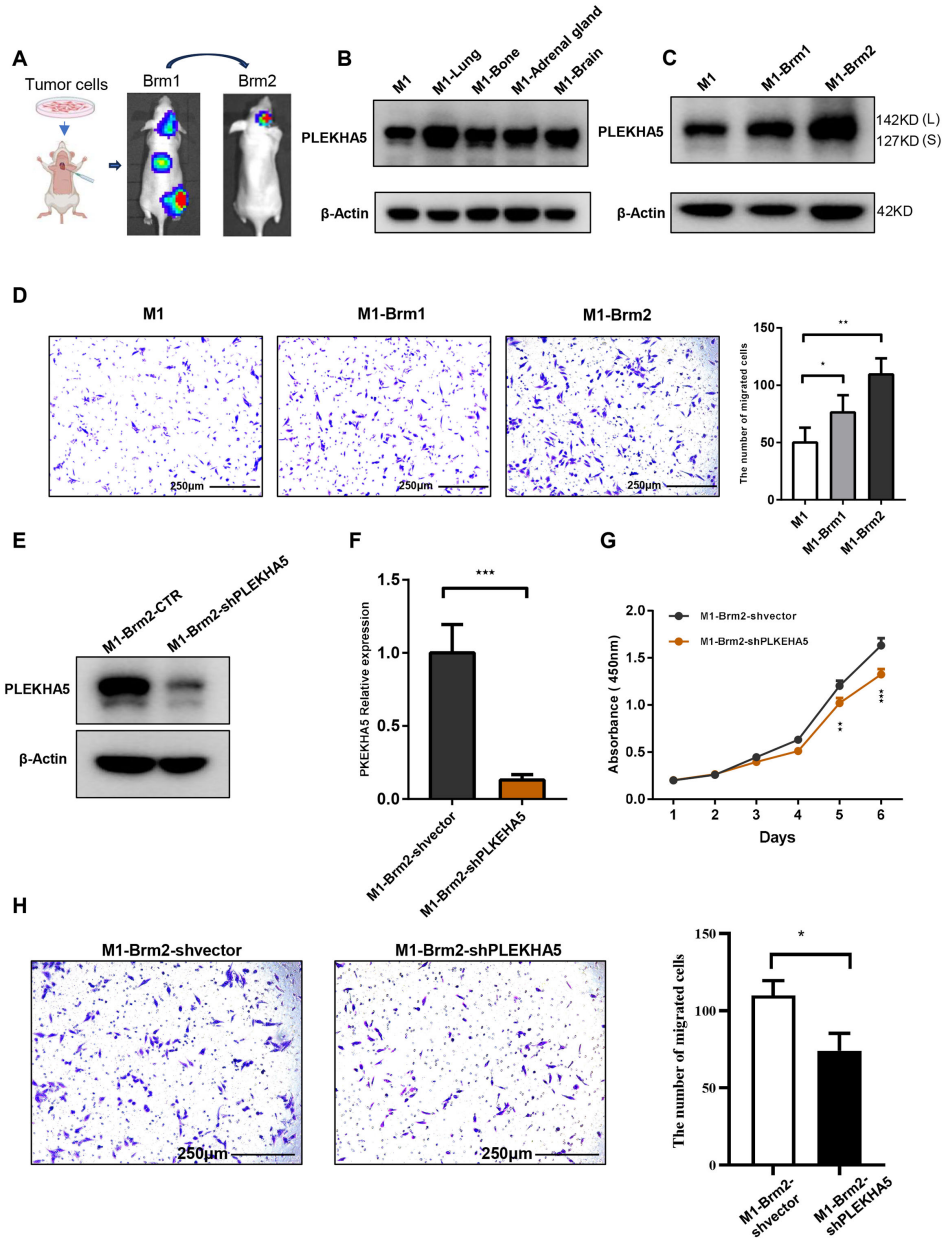
PLEKHA5 promotes brain metastasis and enhances migratory capacity of melanoma cells. **(A)** Schematic of left ventricular injection model tracking melanoma cell metastasis to the brain. **(B)** PLEKHA5 expression in melanoma metastases across multiple organs. **(C)** PLEKHA5 expression in secondary brain metastases and primary cultures. **(D)** PLEKHA5 expression enhances migratory capacity of melanoma cells. **(E–G)**. PLEKHA5 knockdown inhibits the proliferation of M1-Brm2 cells. **(H)** Transwell migration assays of M1-Brm2 cells with PLEKHA5 knockdown. Experiments were performed three times independently with similar results. For panels **B** and **D**, one-way ANOVA (n=3) was used. For panels **F**, **G**, and **H**, t-test (n=3 or n=6) was performed. *P<0.05, **P<0.01, ***P<0.001.

To explore the role of PLEKHA5 in the regulation of melanoma brain metastasis, we initiated our investigation with a series of left ventricular injections in mice. This approach allows for the direct assessment of metastatic potential and the opportunity to track the progression of cancer cells from the primary injection site to the brain, which is a common site for melanoma metastasis (**Figure 3A**). Initially, we established a melanoma cell line by primary culturing the clinic tissue of brain metastases, named as JUN-M1 cell (labeled as M1 for short subsequently in the manuscript and figures).

The left ventricular injection of M1 cell had established brain metastases by four weeks post-injection. The expression of PLEKHA5 increased in metastases of multiple organs such as lung, bone, adrenal gland, and brain (**Figure 3B**). We then isolated the primary brain metastases formed by M1 cells from the mice and established a primary culture, which we designated as M1-Brm1. Subsequently, we reintroduced M1-Brm1 cells into the left ventricle of mice to spontaneously form secondary brain metastases. Four weeks after the second injection, M1-Brm1 cells successfully developed a second round of brain metastases. These were harvested from the mice and cultured to establish a second primary culture, which we named M1-Brm2. We utilized Transwell chamber migration assays to demonstrate that the elevated expression of PLEKHA5 in melanoma cells post-brain metastasis was associated with a marked enhancement in migratory capacity (**Figures 3C, D**). Collectively, our results indicate that PLEKHA5 is highly expressed in melanoma cells with a propensity for brain metastasis and plays a promotional role in the formation of melanoma brain metastases in mice.

### PLEKHA5 was associated to malignancy of brain metastatic melanoma

**Figure 4:**
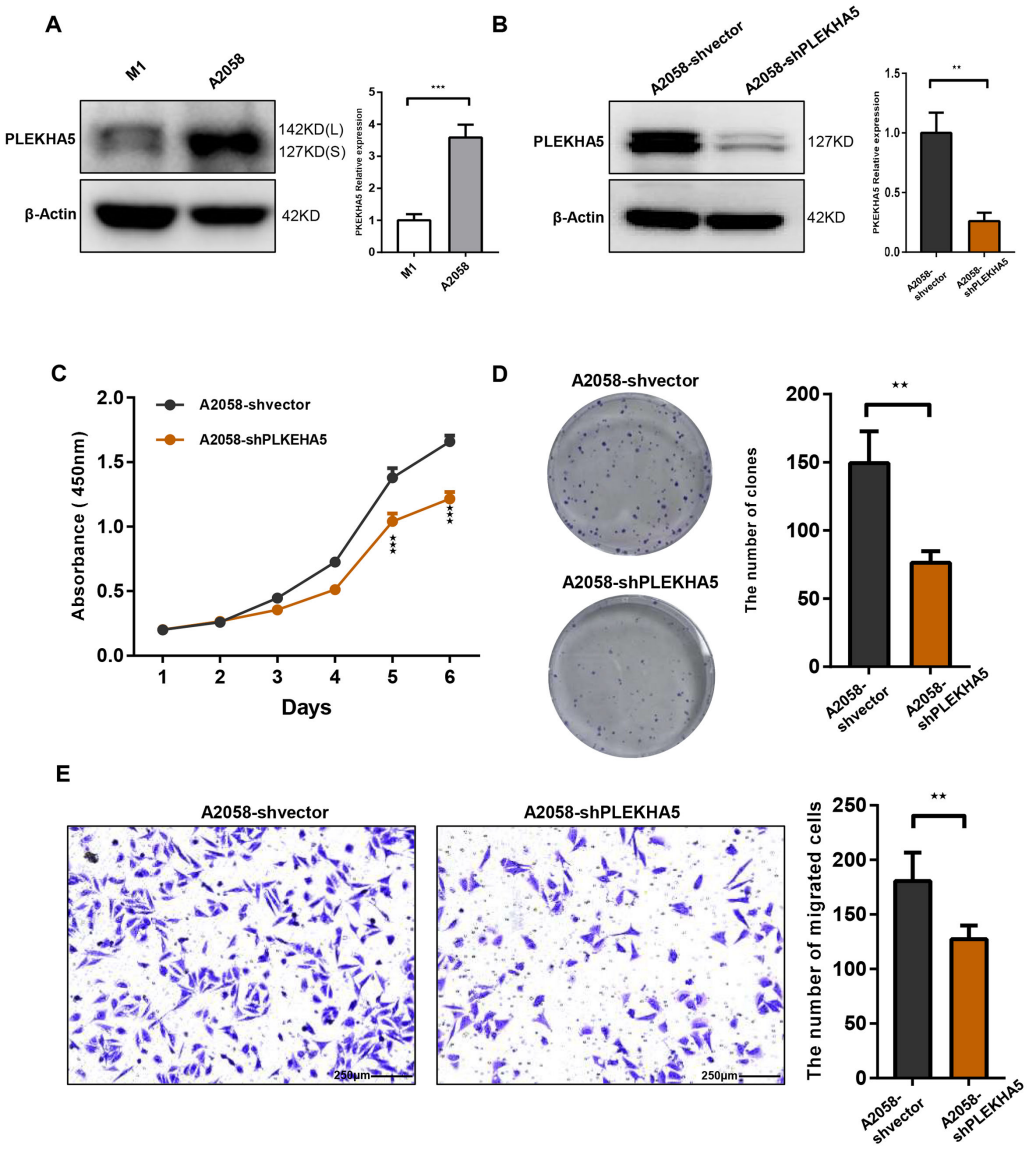
PLEKHA5 knockdown inhibits the proliferation and migration of A2058 melanoma cells. **(A)** Comparison of PLEKHA5 gene expression levels in M1 and A2058 cell lines. **(B)** Protein expression levels of PLEKHA5 in A2058 cells following shRNA-mediated knockdown. **(C)** CCK8 proliferation curves showing a significant inhibition of A2058 cell proliferation upon PLEKHA5 knockdown. **(D)** Colony formation assays demonstrating that PLEKHA5 knockdown significantly inhibits the anchorage-dependent growth and proliferative capacity of A2058 cells. **(E)** Transwell migration assays showing a significant reduction in the migratory capacity of A2058 cells following PLEKHA5 knockdown.Experiments were performed three times independently with similar results. For panels **A**, **B**, and **E**, t-test (n=3) was used. For panel **C**, t-test (n=6) was used. **P<0.01, ***P<0.001.

To investigate the role of PLEKHA5 in the regulation of melanoma brain metastasis, we initiated our study with a series of experiments to assess the proliferative capacity of melanoma cells. We employed the CCK8 assay to monitor the growth and proliferation of melanoma cells. The proliferation curves indicated a significant suppression of M1-Brm2 cell proliferation upon PLEKHA5 knockdown (**Figures 3E–G**). Transwell chamber migration assays indicated a significant suppression of M1-Brm2 cell mobility upon PLEKHA5 knockdown (**Figure 3H**). To confirm the role of PLEKHA5 on melanoma malignancy, we applied the *in vitro* assays on another melanoma cells A2058 that expressed more PLEKHA5 than M1 cells (**Figure 4A**). The CCK8 assay indicated a significant suppression of A2058 cell proliferation upon PLEKHA5 knockdown (**Figures 4B, C**). To further substantiate our findings, we conducted colony formation assays to evaluate the impact of PLEKHA5 on the anchorage-dependent growth and proliferative capacity of melanoma cells. Our observations revealed that PLEKHA5 knockdown markedly inhibited the colony formation of A2058 cells (**Figure 4D**). Collectively, these results suggest that downregulation of PLEKHA5 can inhibit the proliferation of melanoma cells.

We further investigated the impact of PLEKHA5 knockdown on the migratory capacity of melanoma cells. Utilizing the Transwell chamber migration assay, we observed a significant reduction in the number of A2058 cells traversing the Transwell chamber following PLEKHA5 knockdown (**Figure 4E**), indicating that PLEKHA5 knockdown can inhibit the migration of melanoma cells A2058.

### PLEKHA5-L increased the proliferation and migration of melanoma cells

**Figure 5:**
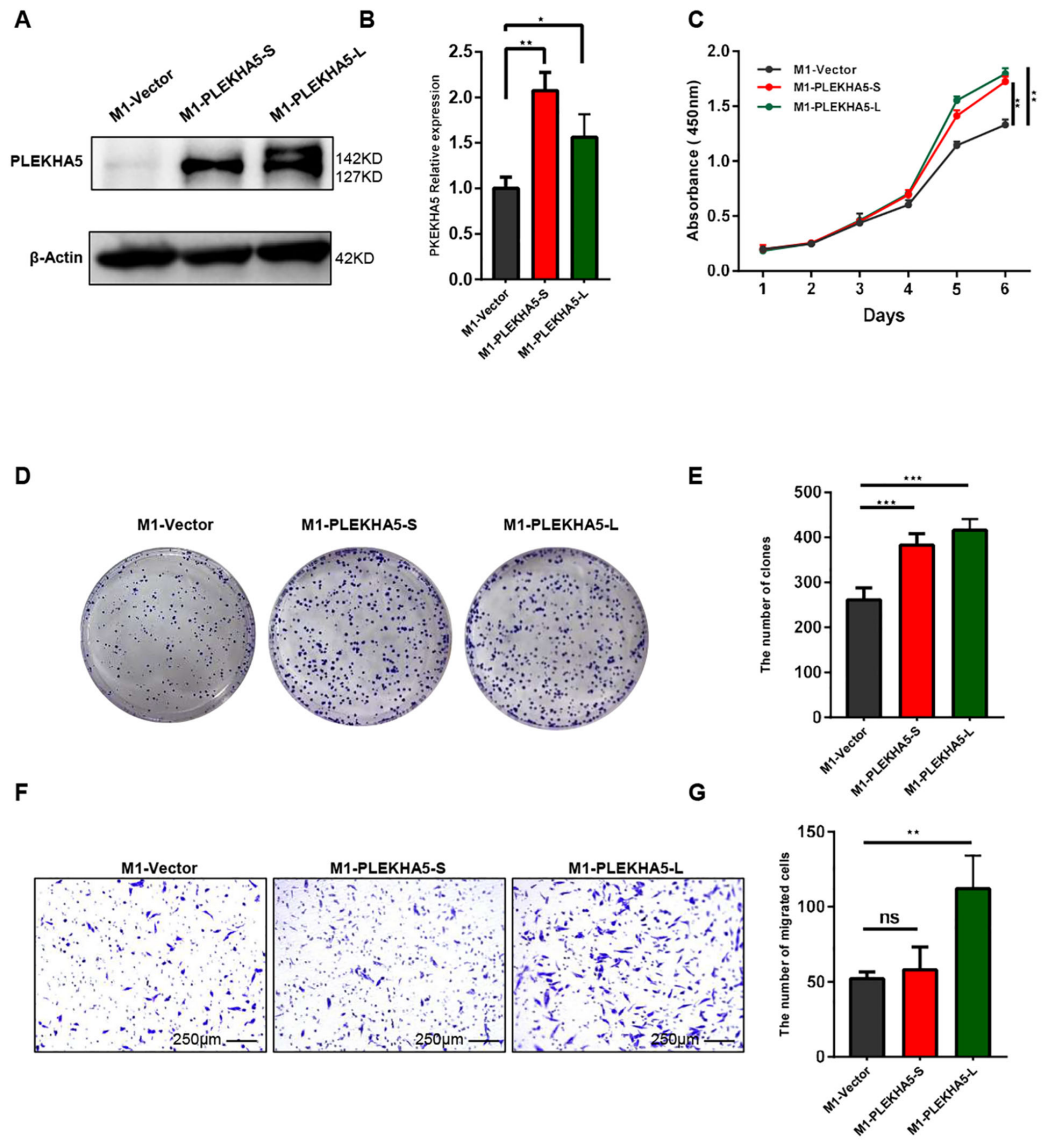
PLEKHA5-L enhances the metastasis of melanoma cells. **(A, B)** Western blot analysis showing overexpression of PLEKHA5-S (127 kDa) and PLEKHA5-L (142 kDa) in M1 cells. **(C)** CCK8 proliferation curves comparing M1 wild type(vector), M1-PLEKHA5-S, and M1-PLEKHA5-L cells, indicating enhanced cell growth in the overexpressing groups. **(D, E)** Colony formation assay showing that both PLEKHA5-S and PLEKHA5-L overexpression significantly increases the proliferative capacity of M1 cells. **(F, G)** Transwell migration assay showing that overexpression of PLEKHA5-L significantly increases the migratory capacity of M1 cells, whereas PLEKHA5-S does not significantly affect migration. Experiments were performed three times independently with similar results. For panels **A** and **B**, t-test (n=3) was used. For panel **C**, one-way ANOVA (n=6) and for panels **D**, **E**, **F**, and **G** (n=3) was used. *P<0.05, **P<0.01, ***P<0.001. ns, not significant.

PLEKHA5 primarily has two major splice isoforms, PLEKHA5-S and PLEKHA5-L. Whether these two subtypes have different effects on the growth and metastasis of melanoma remains unreported. To explore the specific functions of PLEKHA5-S and PLEKHA5-L, we overexpressed each separately in M1 cells. Initially, we overexpressed the PLEKHA5-S (3351 bp in length) and PLEKHA5-L (3849 bp in length) fragments (**Figures 5A, B**). The overexpression cell lines for PLEKHA5-S (127 KD) and PLEKHA5-L (142 KD) were successfully established.

To explore the impact of overexpressing PLEKHA5-S and PLEKHA5-L on the proliferative capacity of melanoma cells, we first conducted a CCK8 assay to monitor the growth and proliferation of melanoma cells. The proliferation curves indicated that the overexpression of both PLEKHA5-S and PLEKHA5-L promoted the proliferation of M1 cells (**Figure 5C**). To further substantiate our experimental results, we then performed colony formation assays to demonstrate the effects of PLEKHA5-S and PLEKHA5-L on the anchorage-dependent growth and proliferative capacity of melanoma cells. The overexpression of both PLEKHA5-S and PLEKHA5-L significantly enhanced the colony formation of M1 cells (**Figures 5D, E**). These results suggest that the overexpression of PLEKHA5-S and PLEKHA5-L in the primary melanoma cells M1 can both promote the proliferation of the primary melanoma cells M1.

Next, we further investigated the impact of overexpressing PLEKHA5-S and PLEKHA5-L on the migratory capacity of melanoma cells. Through the Transwell chamber migration assay, we found that among M1 cells overexpressing PLEKHA5-S and PLEKHA5-L, the number of M1-PLEKHA5-L cells that traversed the Transwell chamber significantly increased, while the number of M1-PLEKHA5-S cells that traversed the Transwell chamber did not significantly change (**Figures 5F, G**).

These results indicate that among the two major splice isoforms of PLEKHA5 (PLEKHA5-S and PLEKHA5-L), only PLEKHA5-L can enhance the migration of melanoma cells. The increased expression of PLEKHA5-L might be important for the brain metastasis of melanoma.

## RNA-seq reveals PLEKHA5-L’s multifaceted impact on melanoma brain metastasis

**Figure 6:**
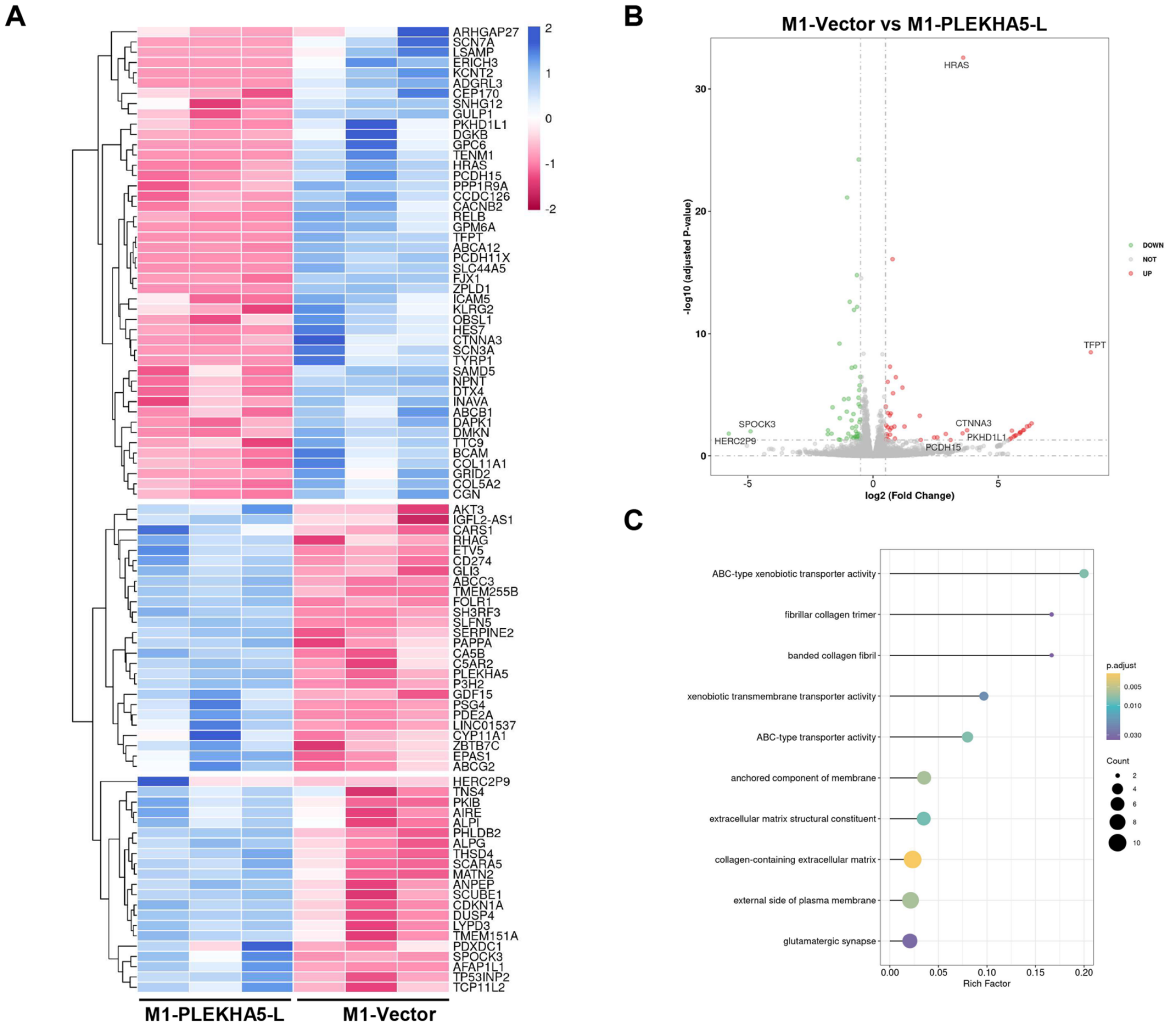
Molecular Mechanism of PLEKHA5-L Regulation in Melanoma Cells. **(A)** Heatmap showing differential gene expression between M1 cells overexpressing PLEKHA5-L and control vector. **(B)** Volcano plot illustrating the significant changes in gene expression upon PLEKHA5-L overexpression. **(C)** Key genes upregulated in PLEKHA5-L overexpressing cells, including HRAS, AKT3, CD274 (PD-L1), and SERPINE2, with potential roles in tumor progression, immune evasion, and metastasis. KEGG pathway analysis indicates increased activity of ATP-binding cassette (ABC) transporters, suggesting potential implications for chemoresistance.

To study the molecular mechanism of PLEKHA5-L on regulating melanoma cells, we applied RNA-seq on M1 cells that expressed PLEKHA5-L and control vector respectively. The heatmap and volcano plot shown the difference of gene expression (**Figures 6A, B**). HRAS and AKT3 expression increased in PLEKHA5-L overexpressed cells. The increased activation of Akt3 plays an important role in the development of more aggressive tumors, and the detection of Akt3 expression and activity in metastatic melanoma indicates that it is upregulated in 43%-60% of advanced metastatic melanoma (15). CD274 (also called PD-L1) expression also increased in PLEKHA5-L overexpressed cells. The upregulation of PD-L1 expression in tumor cells and the upregulation of PD-1 expression on tumor-infiltrating lymphocytes are involved in the related immune inhibitory signal transduction. The activation of the PD-1/PD-L1 signaling pathway inhibits the proliferation and activation of CD4+ T cells and CD8+ T cells, suppresses the expression of cytokines, alters the tumor microenvironment, weakens the body’s surveillance and clearance ability against tumor cells, and promotes tumor cells to evade immune surveillance and killing by the body (16). KEGG analysis indicated that PLEKHA5 increased the activity of ATP-binding cassette (ABC) transporters (**Figure 6C**). ABC transporters are associated the drug resistance, which suggest PLEKHA5-L might also promote the chemoresistance of melanoma.

## Discussion

Melanoma is the deadliest form of skin cancer, and the brain is a common site for metastatic spread of melanoma. It is estimated that 40-50% of stage IV melanoma patients will eventually develop clinically detectable brain metastases (1–3). However, the molecular mechanisms underlying melanoma brain metastasis remain unclear, and there is an urgent need for new molecular markers as targets for prevention and treatment.

PLEKHA5 is a member of the pleckstrin homology domain-containing protein family A, primarily involved in signal transduction, cytoskeletal rearrangement, membrane protein targeting, and vesicular transport (9). PLEKHA5 exhibits multiple variants in mice and humans, with the long form (PLEKHA5-L) and the short form (PLEKHA5-S) being the two main variants, both of which play a role in normal brain development (10).

Published studies have indicated that PLEKHA5 plays different roles in breast cancer, melanoma, and gastric cancer, and the reasons for the functional differences of PLEKHA5 in different tumors are not yet clear. One hypothesis is that PLEKHA5 splice isoforms (PLEKHA5-L and PLEKHA5-S) may have opposing functions in tumor formation and metastasis (13). Further research is needed to elucidate the roles of different PLEKHA5 variants in tumorigenesis.

Previous reports have shown that PLEKHA5 is highly expressed in melanoma brain metastases and promotes the growth of melanoma brain metastases (11, 12). However, it is currently unclear which form of PLEKHA5 plays a role in the formation of melanoma brain metastases. In this study, we found that both PLEKHA5-L and PLEKHA5-S promote the proliferation of melanoma cells, but only PLEKHA5-L promotes the migration of melanoma cells. By establishing a melanoma brain metastasis model through intracardiac injection of melanoma cells, and subsequent primary culture of the metastatic foci, we discovered that melanoma brain metastasis cells have significantly higher expression of PLEKHA5-L compared to wild-type cells, and the migration ability of melanoma brain metastasis cells is also significantly increased. We further confirmed that PLEKHA5-L affects the growth and migration of melanoma brain metastasis cells. Although our analysis of public single-cell sequencing datasets revealed upregulation of PLEKHA5 in melanoma patients with brain metastasis, the elevated expression of the PLEKHA5-L isoform requires further validation using advanced splicing analysis technologies capable of resolving isoform-specific contributions in heterogeneous tumor contexts (17). These findings suggest that PLEKHA5-L plays an important role in melanoma brain metastasis.

To explore the molecular mechanisms, we performed high-throughput sequencing on melanoma cells overexpressing PLEKHA5-L and their control cells. Through differential gene analysis, we found that the expression of genes such as Akt3, CD274 (PD-L1), SH3RF3, SERPINE2, and AFAP1L1 also increased with the upregulation of PLEKHA5-L. Cancer metastasis is a multi-step process involving complex and highly coordinated interactions between tumor cells and the constantly changing host microenvironment. Increased activation of Akt3 plays an important role in the development of more aggressive tumors, and detection of Akt3 expression and activity in metastatic melanoma has shown elevated expression in 43-60% of advanced metastatic melanoma cases (15). In our previous study, downregulation of PLEKHA5 by shRNA sensitived melanoma cells to the AKT inhibitor. In humans, tumor antigens expressed on tumor tissue, although recognized by host T cells, are not necessarily cleared. One reason is the upregulated expression of PD-L1 and PD-1 in the tumor microenvironment. The upregulation of PD-L1 in tumor tissues (such as lymphoma, choriocarcinoma, melanoma, esophageal cancer) and PD-1 on tumor-infiltrating lymphocytes participates in related immune inhibitory signal transduction. Activation of the PD-1/PD-L1 signaling pathway inhibits the proliferation and activation of CD4+ T cells and CD8+ T cells, suppresses the expression of cytokines, alters the tumor microenvironment, weakens the body’s surveillance and clearance capabilities against tumor cells, and promotes tumor cells to evade immune surveillance and killing (16). SERPINE2 has been found to be associated with vasculogenic mimicry in breast cancer, and the formation of vasculogenic mimicry can enhance the ability of tumor cells to metastasize to distant sites (18). Whether SERPINE2 promotes vasculogenic mimicry in melanoma brain metastasis needs further validation. However, studies have shown that SERPINE2 promotes the metastasis of melanoma cells through the GSK-3β signaling pathway both *in vitro* and *in vivo* (19). AFAP1 has been shown to affect the dynamic changes of actin filament cross-linking, and AFAP1 may also play a role in actin cytoskeletal remodeling, thereby regulating cell shape and movement (20). The upregulation of genes related to tumor invasion, metastasis, and cytoskeletal remodeling following PLEKHA5-L overexpression may be one of the reasons for the occurrence of melanoma brain metastasis.

On conclusion, we analyzed the brain metastatic melanoma at single cell level and found PLEKHA5 expression was upregulated, which was subsequently validated in nude mice model. PLEKHA5-L had the potential for melanoma migration and proliferation by upregulating oncogenes such as HRAS, AKT3 etc. PLEKHA5-L also upregulated expression of PD-L1and ABC transporters that were associated to therapy resistant. PLEKHA5-L was potential therapeutic target for metastatic melanoma.

## Data Availability

The original contributions presented in the study are included in the article/**Supplementary Material**, further inquiries can be directed to the corresponding author/s.
